# TiO_2_@Cu_2_O n-n Type Heterostructures for Photochemistry

**DOI:** 10.3390/ma14133725

**Published:** 2021-07-02

**Authors:** Anita Trenczek-Zajac, Joanna Banas-Gac, Marta Radecka

**Affiliations:** 1 Department of Inorganic Chemistry, Faculty of Materials Science and Ceramics, AGH University of Science and Technology, al. A. Mickiewicza 30, 30-059 Krakow, Poland; radecka@agh.edu.pl; 2 Institute of Electronics, Faculty of Computer Science, Electronics and Telecommunications, AGH University of Science and Technology, al. A. Mickiewicza 30, 30-059 Krakow, Poland; jbanas@agh.edu.pl

**Keywords:** photoelectrocatalysis, TiO_2_/Cu_2_O heterostructures, hydrogen, photoelectrode, n-n heterojunction

## Abstract

A TiO_2_@Cu_2_O semiconductor heterostructure with better photochemical response compared to TiO_2_ was obtained using an electrochemical deposition method of Cu_2_O on the surface of TiO_2_ nanotubes. The choice of 1D nanotubes was motivated by the possibility of achieving fast charge transfer, which is considered best suited for photochemical applications. The morphology and structural properties of the obtained heterojunction were determined using standard methods —SEM and Raman spectroscopy. Analysis of photoelectrochemical properties showed that TiO_2_@Cu_2_O heterostructures exhibit better properties resulting from an interaction with sunlight than TiO_2_. A close relationship between the morphology of the heterostructures and their photoproperties was also demonstrated. Investigations representing a combination of photoelectrochemical cells for hydrogen production and photocatalysis—photoelectrocatalysis—were also carried out and confirmed the observations on the photoproperties of heterostructures. Analysis of the Mott–Schottky plots as well as photoelectrochemical measurements (I_ph_-V, I_ph_-t) showed that TiO_2_ as well as, unusually, Cu_2_O exhibit n-type conductivity. On this basis, a new energy diagram of the TiO_2_@Cu_2_O system was proposed. It was found that TiO_2_@Cu_2_O n-n type heterostructure prevents the processes of photocorrosion of copper(I) oxide contained in a TiO_2_-based heterostructure.

## 1. Introduction

The illumination of the semiconductor with light characterized by hν > Eg is related to the creation of electrons (e^−^) in the conduction band and electron holes (h^•^) in the valence band. After the creation of the carriers, the separation of the opposite charges in an electric field should occur. The charge carriers can be used indirectly to drive a chemical reaction (photocatalysis or photoelectrolysis) [1,2,3,4]. The difference between photoelectrolysis and photocatalysis is the subsequent mode of action of the photogenerated electron–hole pair, which can take part in the redox reaction in two basic configurations: the particulate system and the photoelectrochemical cell PEC [3]. In a photocatalytic process, the semiconductor powders are suspended in a solution and the photogenerated holes and electrons react with chemical species such as H_2_O, OH^−^, or O_2_ to produce hydroxyl radicals (^•^OH), superoxide radical anions (O_2_^•−^), and H_2_O_2_, which contribute to the decomposition of adsorbed molecules at the semiconductor surface. The photoelectrolysis is conducted in PEC. For example, an n-type semiconductor acts as a photoanode and a second electrode is metallic (when n-type semiconductor photoanode is used). In photoelectrolysis of water, holes oxidize water, which results in oxygen generation at the photocatalyst surface. The electrons are transported over an external circuit to the cathode, where hydrogen is generated via the reduction of water. The photoelectrocatalysis process, which combines both electrolysis and photocatalysis, is an excellent example of the ability to delay the recombination of electron-hole pairs, increasing the lifetime of the photocarriers. The photogenerated holes act as strong oxidizing species, whereas e^−^ play a role of potential reductor. The photogenerated holes act as strong oxidizing species, whereas e^−^ play a role of potential reductor [5]. The last two decades have seen a significant increase in the number of publications on the treatment of organic pollutants in wastewater [6,7,8,9]. Titanium dioxide (TiO_2_) is the preferred material for photoanodes in photoelectrocatalytic applications [3,6,8,9,10], although WO_3_, ZnO, and other materials are also proposed [3,6,8,9]. Titanium dioxide, TiO_2_, has been the first material applied as a photoanode in the PEC for photoelectrolysis of water into hydrogen and oxygen [11]. TiO_2_ is one of the most suitable candidates for photoanodes due to its high resistance to corrosion, stability, and negative flat band potential. However, the band-gap of TiO_2_ is in the order of 3 eV and, as a consequence, the absorption of sunlight is hindered. Several methods that could improve the photoresponse of TiO_2_ have been considered [2,3,10,12,13,14,15]. The strategies are focused on improving the performance of photocatalysts such by doping, co-doping, band-gap engineering, co-catalyst decoration, heterostructures junction formation, or modification of the microstructure and morphology. Among these propositions, the use of nanomaterials (0D, 1D, 2D, 3D) and heterostructures of metal oxide semiconductors based on a heterojunction with different relative positions of the edges of the conduction and valence bands deserves special attention. From the point of view of achieving fast charge transfer, 1D nanorods and nanotubes are considered the best suited for this purpose. Nanotubes present a natural means for the fast channeling of charge carriers [10,16,17]. In the literature, three classes of heterojunctions depending on the direction of charge transfer are reported [15,18]. For Type I, the conduction band minimum of one semiconductor (CB_1) is below CB_2 of the other one, and its valence band maximum (VB_1) is above VB_2. Type II occurs when both the conduction band minimum CB_1 and valence band maximum VB_1 of one semiconductor are below those of the second one. Type III is similar to type II, but with much bigger energetic separation between the band edges of the two semiconductors. In the case of the photoelectrocatalytic process, only the type of configuration where electrons can be transferred via an external circuit from the photoanode to the cathode, is suitable.

The efficiencies η of TiO_2_ in various forms (i.e., nanowires, nanotubes, thin layers) and heterostructures composed of TiO_2_ and different narrow and wide band-gap semiconductors are presented in Table 1. The coefficient η was calculated at the biased potential V_B_ = 0 and 0.5 V based on the photocurrent vs. potential characteristics. The higher the potential difference is, the higher efficiency is reached. However, it should be emphasized that the “green” approach expects high efficiency of PEC with zero external potential difference. Among the TiO_2_ photoanodes, the highest efficiency equal to 1.9 was achieved at 0.5 V for a thin layer of TiO_2_. Relatively high values of η are also attributed to the TiO_2_@MoS_2_ system, but their interpretation is difficult due to the lack of information about V_B_ at which they were obtained. In the case of TiO_2_ nanotubes modified with tin dioxide, the range of obtained efficiencies is wide (0.21–2.12%), which results from differences in the morphology and chemical properties of these materials. The modification of TiO_2_ nanowires with copper(I) oxide also results in an increase in η; however, the values are not too high.

Efficiency η (solar-to-chemical energy conversion efficiency) is expressed as follows:

(1) $$\eta =\frac{I_{\mathrm{ph}}\;\left(V_{r\;}{-\;V}_{B}\right)}{P}$$

where I_ph_—photocurrent density in the circuit while the electrode is illuminating $\left(\frac{mA}{cm^{2}}\right)$, V_r_—redox reaction potential (V_r_ = 1.23 V vs. NHE), V_B_—biased potential (V), and P—light power density $\left(\frac{mW}{cm^{2}}\right)$.

Copper(I) oxide (Cu_2_O) is a promising narrow band-gap semiconductor for photoelectrochemical applications due to its relative position of energy bands and band-gap energy of 2.0–2.5 eV [26,27,28,29]. However, the poor stability and the fast electron–hole recombination are a serious limitation for the application of Cu_2_O in photoelectrocatalysis. On the other hand, there are several approaches that can be used to stabilize and improve the efficiency of Cu_2_O as a photoelectrode. One of them is utilizing a suitable n-type semiconductor to combine with Cu_2_O. The formation of a junction between two semiconductors is favorable for inhibiting the fast recombination of photocharges. In addition, Cu_2_O joined with a wide band-gap semiconductor prevents the narrow band-gap semiconductor from photocorrosion.

In this work, we have studied titanium dioxide nanotubes (TiO_2_-NT) modified by Cu_2_O with the ultimate aim of determining band-gap alignment of TiO_2_-NT@Cu_2_O heterojunction. The aim of this research is to demonstrate the effect of TiO_2_-NT@Cu_2_O heterostructures, in particular, on the performance of the process of photoelectrolysis and photoelectrocatalysis in PEC cells for hydrogen generation and the decomposition of organics dyes.

## 2. Materials and Methods

### 2.1. Materials

Acetone (analytically pure), isopropanol (analytically pure), glycerol (analytically pure), Na_2_SO_4_ (analytically pure), CuSO_4_ (analytically pure), NaOH (pure), and methylene blue were purchased from Avantor Performance Materials (Gliwice, Poland). Lactic acid (88%, analytically pure) was acquired from Chempur (Piekary Slaskie, Poland). NH_4_F (≥98.0%, ACS reagent), Ti foil (0.127 mm, 99.7%), tert-butanol (≥99.0%, ACS reagent), ethylenediaminetetraacetic acid disodium salt (99.0–101.0%, ACS reagent) and p-benzoquinone (≥99.5%, HPLC) were purchased from Sigma-Aldrich (Saint Louis, MO, USA). Argon (pure) was purchased from Air Liquide (Paris, France).

### 2.2. Samples Preparation

Titanium dioxide nanotubes (TiO_2_-NT) were prepared via an anodization process according to the procedure described in our previous paper [24]. The Cu_2_O electrochemical deposition process was performed in a three-electrode cell: TiO_2_-NT—working electrode, Pt—counter electrode, Ag/AgCl—reference electrode. Before each deposition of Cu_2_O, a fresh electrolyte was prepared consisting of 50 cm^3^ 0.4 M copper(II) sulfate(VI), 12.6 cm^3^ 11.8 M lactic acid. The pH = 12 was established by adding the appropriate volume of 4.0 M NaOH. Electrodeposition was carried out in a solution heated up to a temperature of 60 °C. The potential difference was equal to −0.36 V during the process and the time lasted from 30 to 180 s. After deposition, TiO_2_-NT@Cu_2_O samples were rinsed with deionized water and dried in air at an ambient temperature. Detailed parameters of Cu_2_O deposition process are reported in Table 2.

### 2.3. Characterisation Techniques

Scanning Electron Microscope (SEM) NOVA NANO SEM 200 (FEI EUROPE COMPANY, Hillsboro, OR, USA) was used to observe the surface morphology. Raman spectra were recorded with the use of Witec Alpha 300M+ (Ulm, Germany) equipped with a blue laser (λ = 488 nm) and a confocal microscope with an Epiplan-Neofluar ZEISS (Oberkochen, Germany) lens (magnification of 100×). The photoelectrochemical studies were performed in a three-electrode custom-made photoelectrochemical cell (PEC) in the dark and under the illumination of white light. The photoanode was illuminated by a Xe lamp with a power of 450 W and a power density equal to 100 mW/cm^2^ was used. Three-electrode system consisting of TiO_2_-NT or TiO_2_@Cu_2_O photoanodes which served as a working electrode, a saturated calomel electrode (SCE) as the reference electrode, and Pt-electrode covered with Pt black as the counter electrode were used. Two types of characteristics were measured: current–voltage (I_ph_–V) and current–time (I_ph_–t). The following parameters were analyzed: current density measured in the dark, photocurrent density (I_ph_), flat band potential (V_fb_). The stability of photoelectrodes was evaluated based on photocurrent kinetics. As an alternative method to photocatalysis, photoelectrocatalytic measurements were performed as a combination of photocatalysis and photoelectrolysis. Photoelectrocatalysis was carried out in a system designed for PEC measurements—commercial PECC-2 (ZAHNER-Elektrik GmbH & CoKG, Kronach, Germany). Three electrodes—photoelectrode, Pt, and Ag/AgCl—were immersed in the electrolyte consisting of 0.1 M Na_2_SO_4_ solution and 1.25 · 10^−5^ M methylene blue (MB). A potential difference equal to 1 V was applied to the electrodes during the decomposition process. The external voltage causes the separation of photogenerated charge carriers and thus limits their recombination. The protoelectrocatalytic procedure was as follows. First, determination of the adsorption–desorption equilibrium—the electrolyte with electrodes immersed in it was stirred in the dark for 30 min. Second, the Xe lamp was switched on and a constant potential difference of 1 V was applied. At specified intervals, 4 mL of solution was collected and injected into the measurement cuvettes. The absorbance of the solution was measured in quartz cuvettes using a V-670 UV-VIS-NIR spectrophotometer (Jasco, Tokyo, Japan). After that, the solution was placed back into the PECC-2. For comparison purposes, photoelectrocatalytic decomposition was also performed in the dark and with no external voltage. To determine which reactions occur during the process of photoelectrocatalytic decomposition of MB, three types of scavengers that show affinity to different reactive species were used [30,31,32,33]: *tert*-butanol—hydroxyl radicals, p-benzoquinone—superoxide radical anions, and ethylenediaminetetraacetic acid disodium—electron holes. The type of conductivity was determined with the use of electrochemical impedance spectroscopy based on admittance spectra and the Mott–Schottky plot.

## 3. Results

**Figure 1:**
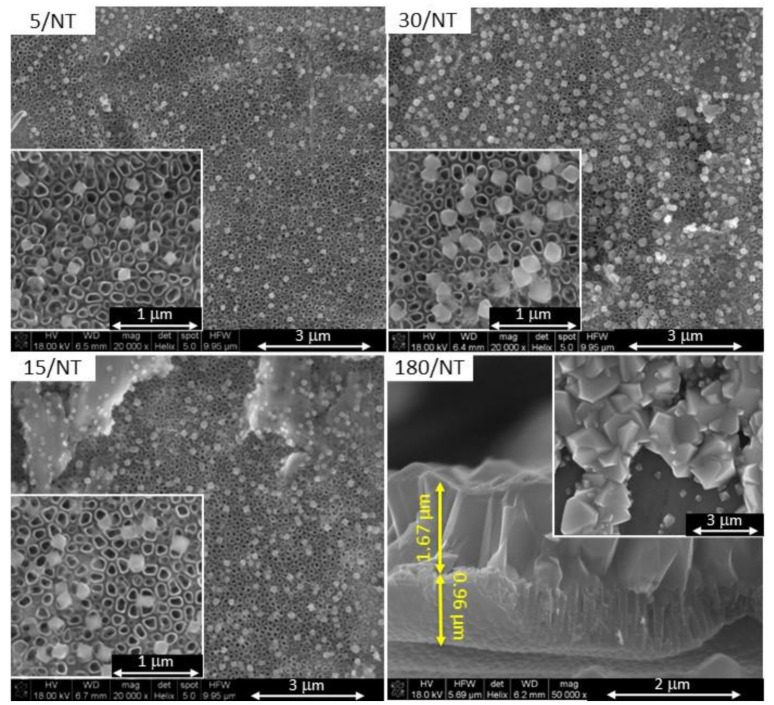
SEM images of TiO_2_-NT@Cu_2_O heterostructures obtained by electrodeposition method in a solution of pH = 12. Deposition parameters: U = −0.36 V, t = 5–180 s.

SEM surface and cross-section images for TiO_2_-NT and TiO_2_-NT@Cu_2_O system prepared in the 5–180 s electrodeposition process are presented in Figure 1 and Figure S1. The sample obtained during 5 s deposition is characterized by Cu_2_O crystals of the smallest size, not exceeding 100 nm. An increase in deposition time up to 30 s slightly increases the crystal sizes up to approximately 300 nm and their quantity. On the other hand, electrodeposition lasting for 180 s leads not only to the complete coverage of the surface of the nanotubes, but also to a significant increase in the size of Cu_2_O crystals ranging from 0.2 to 3.0 μm. The thickness of the Cu_2_O layer is 1.67 μm.

**Figure 2:**
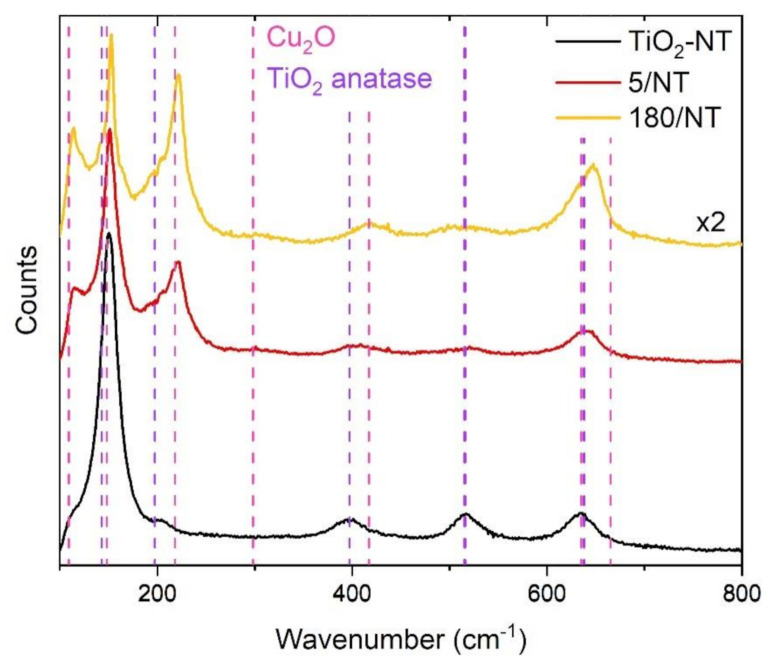
Raman spectra of TiO_2_-NT and TiO_2_-NT@Cu_2_O heterostructures obtained after 5 and 180 s of electrodeposition.

Figure 2 shows Raman spectra of TiO_2_ nanotubes and selected TiO_2_-NT@Cu_2_O heterostructures. The spectrum of titanium dioxide nanotubes displays five Raman modes: 150, 203, 395, 516, and 635 cm^−1^. All of them can be assigned to the TiO_2_ anatase polymorph [34]. After the electrodeposition of Cu_2_O, as expected, the modes arising from TiO_2_ are widened due to partial overlap with new modes derived from copper(I) oxide. What is more, as the prolongation of the electrodeposition time from 5 to 180 s results in a complete coverage of the surface of nanotubes by Cu_2_O (see Figure 1), Cu_2_O Raman modes in the spectrum become better pronounced.

**Figure 3:**
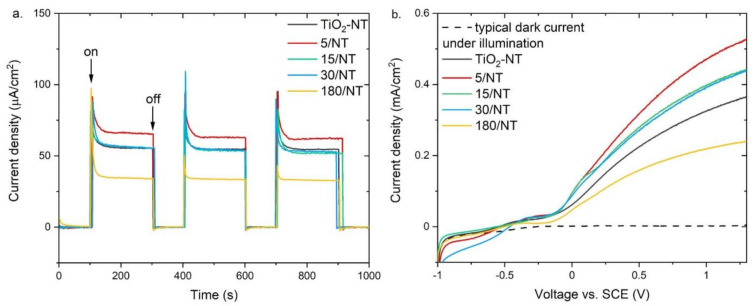
Photoelectrochemical characteristics of TiO_2_-NT and TiO_2_-NT@Cu_2_O anodes: (**a**) current-time and (**b**) current-voltage curves.

Photoelectrochemical properties of TiO_2_-NT@Cu_2_O heterostructures were determined based on the results of current–time and current–voltage measurements. First of all, the tested photoelectrodes, both TiO_2_-NT and heterostructures, demonstrate stability under photoelectrochemical measurement conditions. Figure 3a shows the changes in the kinetics of the photocurrent induced by sudden switching on and off the light illuminating the photoanode. Comparison of the results obtained for TiO_2_ nanotubes and TiO_2_-NT@Cu_2_O heterostructures reveals that the shortest deposition time of copper(I) oxide on the titanium dioxide surface leads to the increase in the photocurrent density of ca. 20%. On the other hand, the longest time of Cu_2_O electrodeposition results in a nearly two-fold decrease in I_ph_ compared to TiO_2_-NT. Figure 3b presents the I_ph_–V characteristics of the PEC cell. Current–voltage curves take a typical shape for a n-type semiconductor photoanode. Heterostructures prepared in the process of deposition lasting no longer than 30 s allowed us to obtain photocurrent density values higher than that for TiO_2_ nanotubes. The consequence of the longest electrodeposition (180 s) is the noticeable reduction in I_ph_ values. The ratio of photocurrent density obtained for heterostructural photoanodes (I_struct_) was also compared to that for TiO_2_ nanotubes (I_TiO2-NT_). I_struct_/I_TiO2-NT_ higher than one means that the photocurrent density for heterostructural photoanodes is higher than for TiO_2_-NT. The flat band potential is negative for all electrodes and is equal to −0.54 ± 0.03 V. Efficiencies calculated for the selected TiO_2_@Cu_2_O heterostructures have shown that there is a significant improvement in relation to TiO_2_ nanotubes and equal to 3.27 and 2.32 @ 0.5 V, respectively. What is more, the value it achieves is higher not only compared to TiO_2_ thin film layer, but also compared to that for the TiO_2_@SnO_2_ (Table 1).

Photoelectrochemical properties of photoanodes, i.e., flat band potential, photocurrent density at U = 0 and 1 V, and the I_struct_/I_TiO2-NT_ ratio at U = 0 and 1 V are summarized in Table 3.

There is a morphological requirement for heterostructures that are applicable in photocatalysis and photoelectrochemistry. For such a system to work efficiently, it is necessary to have quadruple points, i.e., places where access to light and electrolytes is provided to all components of the heterostructure remaining in direct contact with each other. If the layer–particles system is regarded, fulfillment of such a requirement is possible when one of the components is dispersed on the surface of the other. At the same time, it is important that there are many such points in the heterostructure to ensure good photoactivity. The results presented above (Table 3) are in conformity with the observations of morphological differences. The requirements for light and electrolyte access for both components of the heterostructure are fulfilled by the sample obtained during a 5 s deposition. Small copper(I) oxide crystals are distributed evenly on the surface of the nanotubes. On the other hand, the complete coverage of the surface of TiO_2_ nanotubes with a layer of Cu_2_O (Figure 1) means that after illumination of the electrode, chemical reactions occur only on the surface of Cu_2_O. Thus, meeting the morphological criterion in the case of heterostructures with a broad-band semiconductor allows for obtaining a photoanode with better properties than TiO_2_ nanotubes.

**Figure 4:**
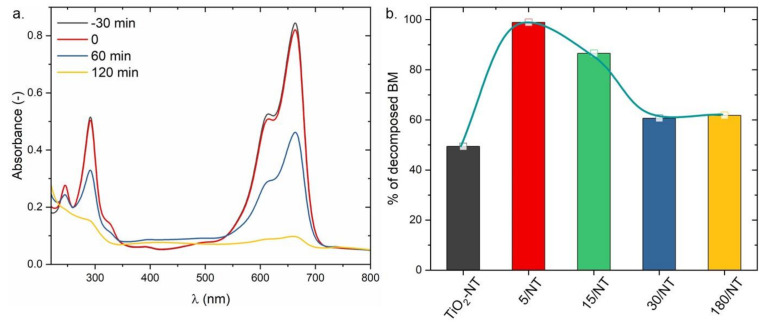
(**a**) Absorption spectra of the MB + Na_2_SO_4_ mixture before and after photoelectrocatalysis of MB obtained for 5/NT heterostructure. (**b**) Comparison of the amount of photoelectrocatalytically decomposed MB after 120 min for different TiO_2_@Cu_2_O heterostructures.

TiO_2_-NT@Cu_2_O heterostructures were also tested in the process of photoelectrocatalysis. The choice of organic dyes is very wide. From among them, methylene blue (MB) belongs to the group of cationic dyes. It is introduced into nature as a water pollutant from the textile industry and is highly toxic and carcinogenic. It is also a model pollutant in the study of photocatalytic activity of semiconductors [35]. That is why the ability of the photoanodes to degrade pollutants was evaluated against MB under sunlight simulated by an Xe bulb and applied a potential difference equal to 1V. Typical spectral dependences of MB absorbance obtained before and during photoelectrocatalytic decomposition are presented in Figure 4a. The time required to establish the adsorption-desorption balance of methylene blue was 30 min. Since the presence of leuco methylene blue was excluded (please refer to Supplementary Materials, Figure S2), the decrease in absorbance can be directly correlated with the decreasing concentration of the partially decomposed dye. Figure 4b shows the percentage of decomposed MB after 60 min of the process for TiO_2_ nanotubes and heterostructures. It is worth noting that all heterostructures show greater activity towards methylene blue decomposition than TiO_2_-NT, even though it removes 49% of MB. The results obtained for TiO_2_-NT@Cu_2_O heterostructures prepared in the electrodeposition process lasting for 5, 15, 30, and 180 s are 99%, 86%, 61%, and 62%, respectively. These results correlate well with the results of photoelectrochemical measurements (see Figure 3b).

**Figure 5:**
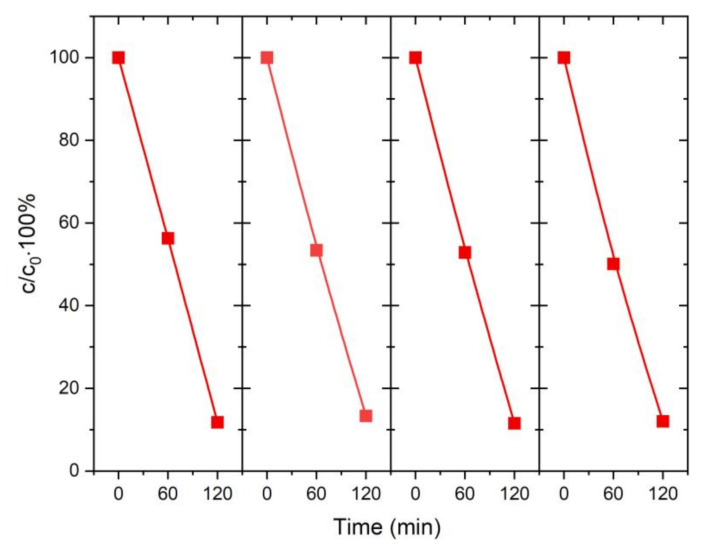
Recycled photoelectrocatalytic test of 5/NT sample.

In terms of the practical application of photocatalysts, stability under measurement conditions is an important issue. Representative TiO_2_@Cu_2_O heterostructure 5/NT was examined in recycle experiments. Photoelectrocatalytic degradation of MB was repeated for four cycles (Figure 5). After every 120 min cycle, almost 90% of the dye was degraded. This indicates that the TiO_2_@Cu_2_O heterostructure is completely stable under those conditions.

**Figure 6:**
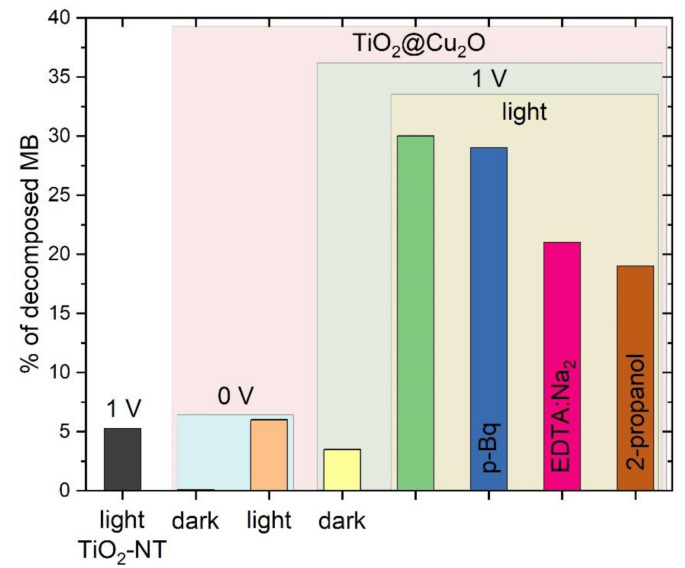
Comparison of the amount of photocatalytically and photoelectrocatalytically decomposed MB after 15 min of process under different conditions: light—illumination with white light, dark—no illumination, 0 V—zero voltage, 1 V—external voltage, pB—p-benzoquinone, EDTA:Na_2_—ethylenediaminetetraacetic acid disodium.

The comparison of the percent of decomposed methylene blue in the process of photocatalytic or photoelectrocatalytic decomposition under different conditions is presented in Figure 6. TiO_2_@Cu_2_O heterostructure was tested both in the dark and under illumination with white light. When the anode was not biased and not illuminated, the percentage of decomposed MB was negligible (0.1%), however, after illumination this value increased to 6.01%. The application of a potential difference of 1 V leads to a sharp increase in the photoactivity of TiO_2_@Cu_2_O and after a time as short as 15 min, 29.92% of MB is degraded. This also means that it is necessary to simultaneously illuminate and polarize the working electrode for the process of photoelectrocatalytic decomposition of methylene blue occurred. For comparison, TiO_2_ nanotubes under the same conditions (1 V, white light) allowed us to obtain only 5.25%. An almost six-fold difference in the percent of decomposed methylene blue clearly indicates an improvement in the photoelectrocatalytic properties of TiO_2_ nanotubes associated with the presence of copper(I) oxide in the TiO_2_@Cu_2_O heterostructure. There are several factors contributing to this effect. First, the range of light absorption of TiO_2_ (UV) is extended by visible light due to the presence of Cu_2_O in the heterostructure. Second, recombination of charge carriers is prevented through the separation of electrons and holes between components of the heterostructure. Similar results regarding the TiO_2_@Cu_2_O system were described by Ma et al. [36]. In their study, they used trichlorophenol as a model for organic pollutant. They postulated that the improved photoactivity of TiO_2_@Cu_2_O in comparison to bare TiO_2_ results not only from the widened absorption range of light, but also from the fact that TiO_2_ and Cu_2_O formed a type II heterostructure. This confirms our assumptions, as type II heterojunction provides an efficient separation of charge carriers between heterostructure elements. Additionally, in the case of photoelectrocatalysis, this is supported by the external voltage.

The process of photoelectrocatalytic decomposition of organic dyes occurs near the photoanode, which indicates the oxidation process is responsible for the degradation [36,37]. The reactive oxygen species that are formed after excitation of the semiconductor with light are as follows:

(2) $$h^{+}+H_{2}O\;\to \;H^{+}+{}^{●}\;\mathrm{OH}$$

(3) $$e^{-}+O_{2}\to {\;O}_{2}^{-}{}^{●}$$

(4) $${O_{2}}^{●-}+H^{+}\;\to {\mathrm{HO}}_{2}{}^{●}$$

(5) $${{2\;\mathrm{HO}}_{2}}^{●}\;\to H_{2}O_{2}+O_{2}$$

(6) $$H_{2}O_{2}+e^{-}\to {\;}^{●}\mathrm{OH}+{\mathrm{OH}}^{-}$$

However, according to da Silva et al. [38], the process of photoelectrocatalytic decomposition of methylene blue occurs mainly as a result of reactions involving electron holes and hydroxyl radicals. To study which of the reactive species are involved in the decomposition reaction of methylene blue in the system with TiO_2_@Cu_2_O (5/NT) photoanode, photoelectrocatalytic processes were carried out with the use of different scavengers that show affinity to hydroxyl radicals (**2**-propanol), oxygen radicals (p-Benzoquinone), and electron holes (ethylenediaminetetraacetic acid disodium). The results obtained at 1 V and after illumination with white light for 15 min are presented in Figure 6. The elimination of oxygen radicals causes degradation of 29.00% of the dye. This is only a percentage point less than that obtained without scavengers under the same conditions. Removal of ^●^OH and $h^{●}$, on the other hand, allows to eliminate 19.26 and 21.12%, respectively, which is approximately 10 percentage points less than without scavengers. On this basis, it can be concluded that oxygen radicals do not participate in the process of the degradation of methylene blue. Instead, the removal of hydroxyl radicals and electron holes significantly contributes to the reduction in the degradation process. This effect suggests that ^●^OH and $h^{●}$ are the basis of the methylene blue degradation mechanism in the discussed process.

## 4. Discussion

**Figure 7:**
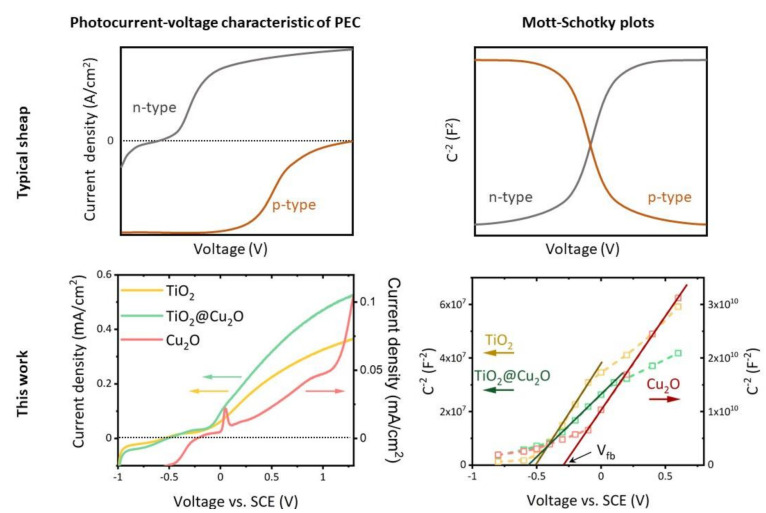
Examples of theoretical and experimental results of selected experimental methods based on photoelectrochemical and electrochemical measurements for determination of the type of conductivity—n- or p-type.

The type of heterojunction is closely related to the type of semiconductor, i.e., p-type or n-type, band-gap energy and band edges position. The n- and p-type conductivity of the photoelectrode based on titanium dioxide nanotubes TiO_2_-NT and copper(I) oxide Cu_2_O were determined on the basis of the analysis of the photocurrent under illumination with white light as a function of the applied bias voltage (V_B_) and Mott–Schottky (M–S) plots. Figure 7 illustrates the methods of analysis of the experimental data for a hypothetical photoanode (n-type) and photocathode (p-type), and the results obtained for the photoelectrodes studied in this work. The M–S plot will possess a negative slope for p-type materials and a positive slope for n-type materials.

In the case of a PEC with a photoanode, the photocurrent characteristic shows a high value of the anodic current, in contrast to the photocathode, which is dominated by the cathodic current. Based on the impedance spectroscopy measurements performed for Cu_2_O, TiO_2_, and TiO_2_@Cu_2_O, Mott–Schottky plots were drawn and are presented in Figure 7 as the C^−2^–V_B_ dependence. The slope of the rectilinear range of C^−2^–V_B_ dependence for the tested photoelectrodes takes positive values. The photocurrent–voltage characteristics also confirm n-type conductivity of the samples. At low temperature, undoped titanium dioxide is well known to be an n-type semiconductor. Although copper(I) oxide is as p-type semiconductor, it was also proved to show n-type conductivity. It is possible to control the type of Cu_2_O conductivity by careful design of the conditions of solvothermal [39] or electrochemical deposition process [40,41].

**Figure 8:**
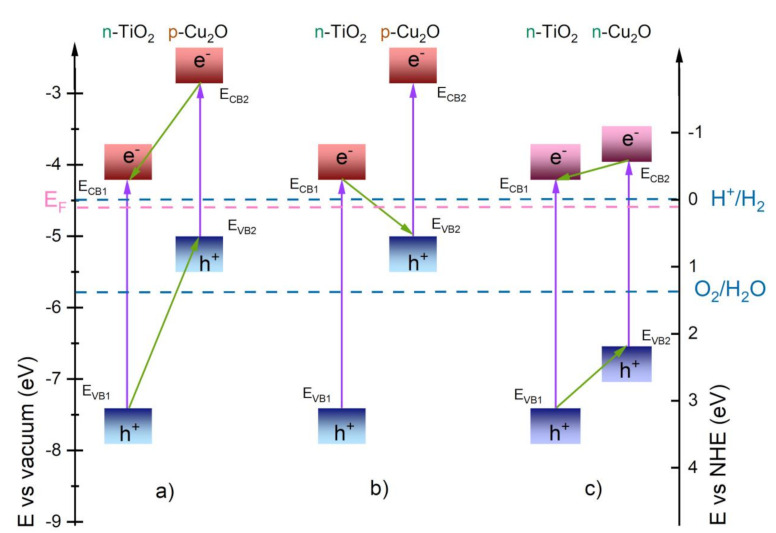
Schematic presentation of possible TiO_2_@Cu_2_O type II heterostructures: p-n heterojunction [42] (**a**), Z-scheme (**b**), and n-n heterojunction (**c**). E_CB_—conduction band of semiconductor, E_VB_—valence band of semiconductor, 1, 2—first and second semiconductor, E_F_—Fermi level.

The current–voltage characteristics of the photoelectrochemical cell with TiO_2_@Cu_2_O photoanode show an increase in photocurrent and solar-to-chemical energy conversion efficiency in relation to the TiO_2_ anode. Similarly, the photoelectrocatalytic degradation of MB reveals an increase in the activity of heterostructures compared to TiO_2_. The nature of Cu_2_O conductivity in the vast majority identified as p-type indicates a possibility of creating a TiO_2_@Cu_2_O p-n system of either type II junction (Figure 8a) or a Z-scheme (Figure 8b). Both configurations of the p-n type TiO_2_@Cu_2_O heterostructure can be considered when the system acts as a photocatalyst. Aguirre et al. [42] indicated that the TiO_2_@Cu_2_O system plays not only the role of a photocatalyst in the CO_2_ reduction reaction, but also prevents Cu_2_O from photocorrosion if the heterostructure acts as a Z-scheme. In this case, the electron holes from the Cu_2_O valence band in the aftermath of recombination with electrons from the TiO_2_ conduction band cannot participate in the process of Cu_2_O photocorrosion. The photoanode based on TiO_2_ modified with copper(I) oxide in a photoelectrochemical cell requires a directed flow of electrons from the anode to the cathode, which means that processes occurring according to the Z-scheme can be difficult. As mentioned before, n-type conductivity of Cu_2_O in the form of layers obtained by the electrodeposition is well known in the literature [43,44]. In this work, n-type conductivity both for Cu_2_O and TiO_2_@Cu_2_O was proved based on both PEC current–voltage characteristics and Mott–Schottky plots. Additionally, taking into account the fact that the flat band potential can be practically identified as the edge of the conduction band, the value of the flat band potential obtained from the M–S plot and I_ph_–V measurements makes it possible to determine the bottom of the conduction band. Flat band potential V_fb_ for Cu_2_O is equal to −0.29 V, whereas for TiO_2_ nanotubes and TiO_2_@Cu_2_O it is comparable and equal to −0.50 and −0.56 V, respectively.

On the basis of the structural, electrical, and photoelectrochemical results, TiO_2_@Cu_2_O heterostructure was proposed to create a heterojunction resulting from the combination of TiO_2_ and Cu_2_O semiconductors in type II n-n configuration (Figure 8c).

In the case of Cu_2_O, the potentials for anodic and cathodic decomposition are located within the band-gap [45], and hence it is necessary to prevent its corrosion. Paracchino et al. named the conditions that should be met in the case of a n-type semiconductor/p-type Cu_2_O heterojunction to not only achieve high efficiency of photoelectrochemical processes but also provide protection against photocorrosion [46]. Among them, conditions of the relative position of the band edges of n-type semiconductor and redox potentials of water; the conduction band edge should be placed above the water reduction level E_red_ (H^+^/H_2_). On the other hand, within its band-gap, there should be no decomposition potentials. The results obtained in this work indicate that in the case of a n-n semiconductor system based on TiO_2_@Cu_2_O, titanium dioxide prevents copper(I) oxide from corrosion by accelerating the transport of electron holes into the electrolyte. This is directly confirmed by the results of repeated photoelectrocatalytic decomposition of methylene blue shown in Figure 5. The percentage of MB decomposed in the experiments conducted did not decrease after four uses. Such a behavior was previously demonstrated by us for TiO_2_@MoS_2_ in photoelectrochemical water decomposition [24].

## 5. Conclusions

Electrodeposition carried out using a mixture of CuSO_4_, lactic acid, and NaOH, with pH = 12, using the difference potential of −0.36 V and short reaction time, allowed to obtain a discontinuous layer of Cu_2_O. This effect is beneficial from the point of view of the photoelectrochemical properties of the resulting Cu_2_O@TiO_2_-NT heterojunction, because it allows absorption in both the UV (TiO_2_) and vis (Cu_2_O) range and electrolyte access to both components of the heterostructure. The obtained Cu_2_O@TiO_2_-NT system was successfully used as a photoelectrode during the alternative to photocatalysis, photoelectrocatalytic decomposition of methylene blue, as a result of which an improvement in properties relative to unmodified titanium dioxide was demonstrated. A new configuration of band-gap alignment for heterojunction of Cu_2_O@TiO_2_-NT was proposed. The analysis of research results indicated that the n-n semiconductor type II heterojunction of Cu_2_O/TiO_2_ had formed. It was found that it prevents the processes of photocorrosion of a semiconductor with a narrower band gap contained in a TiO_2_-based heterostructure.

## Figures and Tables

**Table 1:** Solar-to-chemical energy conversion efficiencies η of TiO_2_ and TiO_2_-based heterostructures. Potential difference was measured relative to the SCE electrode unless stated otherwise.

System	Photoelectrode	Efficiency η (%)		Ref.
		at 0 (V)	at 0.5 (V)	
TiO_2_	TiO_2_ nanorods	0.15 ^1^	–	[19]
	TiO_2_ nanotubes	0.25–1.6	–	[20]
	TiO_2_ thin film	1.65	1.90	[21]
TiO_2_@MoS_2_	TiO_2_ nanofibers	4.7 ^2^		[22]
	TiO_2_@MoS_2_	6.0 ^2^		
TiO_2_@SnO_2_	TiO_2_ nanowires	–	–	[23]
	TiO_2_@SnO_2_	0.21 ^1^	–	
	TiO_2_ nanotubes	0.34	0.39	[24]
	TiO_2_@SnO_2_	0.39–1.61	0.47–2.12	
TiO_2_@Cu_2_O	TiO_2_ nanowires	–	0.14	[25]
	TiO_2_@Cu_2_O	–	0.39	

^1^ Potential difference for Ag/AgCl electrode. ^2^ No information about the potential difference.

**Table 2:** Parameters of TiO_2_-NT@Cu_2_O heterostructures formation process.

Electrode Name	pH	Potential Difference (V)	Time (s)	Substrate
5/NT	12	−0.36	5	TiO_2_-NT
15/NT			15	
30/NT			30	
180/NT			180	

**Table 3:** Photoelectrochemical parameters and conductivity type of TiO_2_-NT and TiO_2_-NT@Cu_2_O heterostructures.

Electrode Name	I_ph_ (μA/cm^2^)		I_hetero_/I_TiO2-NT_		V_fb_ (V)	
	at 0 (V)	at 1 (V)	at 0 (V)	at 1 (V)	Mott-Schottky	I-V ^1^
TiO_2_-NT	61.9	324.9	–	–	−0.50	−0.55
5/NT	94.9	470.7	1.53	1.45	–	−0.52
15/NT	94.5	398.9	1.53	1.23	−0.56	−0.56
30/NT	86.8	392.4	1.40	1.21	–	−0.46
180/NT	50.0	218.9	0.81	0.67	–	−0.50

^1^ For determination method, see [21].

## Data Availability

The data presented in this study are available on request from the corresponding author.

## Supplementary Materials

The following are available online at https://www.mdpi.com/article/10.3390/ma14133725/s1; Figure S1. SEM images of TiO_2_-NT: (**a**) surface and (**b**) cross-section; Figure S2. Absorption spectra of the MB + Na_2_SO_4_ mixture before and after photoelectrocatalysis of MB obtained for 5/NT heterostructure. Inset: position of the band derived from MB (blue) and LMB (orange).

## References

[1] Grätzel M. (2001). Photoelectrochemical cells. Nature.

[2] Marschall R. (2014). Semiconductor composites: Strategies for enhancing charge carrier separation to improve photocatalytic activity. Adv. Funct. Mater..

[3] Li J., Wu N. (2015). Semiconductor-based photocatalysts and photoelectrochemical cells for solar fuel generation: A review. Catal. Sci. Technol..

[4] Thalluri S.M., Bai L., Lv C., Huang Z., Hu X., Liu L. (2020). Strategies for Semiconductor/Electrocatalyst Coupling toward Solar-Driven Water Splitting. Adv. Sci..

[5] Nosaka Y., Nosaka A.Y. (2017). Generation and Detection of Reactive Oxygen Species in Photocatalysis. Chem. Rev..

[6] Garcia-Segura S., Brillas E. (2017). Applied photoelectrocatalysis on the degradation of organic pollutants in wastewaters. J. Photochem. Photobiol. C Photochem. Rev..

[7] Oturan M.A., Aaron J.J. (2014). Advanced oxidation processes in water/wastewater treatment: Principles and applications. A review. Crit. Rev. Environ. Sci. Technol..

[8] Daghrir R., Drogui P., Robert D. (2012). Photoelectrocatalytic technologies for environmental applications. J. Photochem. Photobiol. A Chem..

[9] Bessegato G.G., Guaraldo T.T., de Brito J.F., Brugnera M.F., Zanoni M.V.B. (2015). Achievements and Trends in Photoelectrocatalysis: From Environmental to Energy Applications. Electrocatalysis.

[10] Radecka M., Kusior A., Trenczek-Zajac A., Zakrzewska K. (2018). Oxide Nanomaterials for Photoelectrochemical Hydrogen Energy Sources. Materials for Sustainable Energy.

[11] Fujishima A., Honda K. (1972). Electrochemical photolysis of water at a semiconductor electrode. Nature.

[12] Henderson M.A. (2011). A surface science perspective on TiO_2_ photocatalysis. Surf. Sci. Rep..

[13] Ahmad H., Kamarudin S.K., Minggu L.J., Kassim M. (2015). Hydrogen from photo-catalytic water splitting process: A review. Renew. Sustain. Energy Rev..

[14] Tee S.Y., Win K.Y., Teo W.S., Koh L.D., Liu S., Teng C.P., Han M.Y. (2017). Recent Progress in Energy-Driven Water Splitting. Adv. Sci..

[15] Zhang L., Jaroniec M. (2018). Toward designing semiconductor-semiconductor heterojunctions for photocatalytic applications. Appl. Surf. Sci..

[16] Roy P., Berger S., Schmuki P. (2011). TiO_2_ nanotubes: Synthesis and applications. Angew. Chemie Int. Ed..

[17] Wehrenfennig C., Palumbiny C.M., Snaith H.J., Johnston M.B., Schmidt-Mende L., Herz L.M. (2015). Fast Charge-Carrier Trapping in TiO_2_ Nanotubes. J. Phys. Chem. C.

[18] Low J., Yu J., Jaroniec M., Wageh S., Al-Ghamdi A.A. (2017). Heterojunction Photocatalysts. Adv. Mater..

[19] Sun B., Shi T., Peng Z., Sheng W., Jiang T., Liao G. (2013). Controlled fabrication of Sn/TiO_2_ nanorods for photoelectrochemical water splitting. Nanoscale Res. Lett..

[20] Sun Y., Yan K.P. (2014). Effect of anodization voltage on performance of TiO_2_ nanotube arrays for hydrogen generation in a two-compartment photoelectrochemical cell. Int. J. Hydrogen Energy.

[21] Radecka M., Rekas M., Trenczek-Zajac A., Zakrzewska K. (2008). Importance of the band gap energy and flat band potential for application of modified TiO_2_ photoanodes in water photolysis. J. Power Sources.

[22] Menon H., Gopakumar G., Nair V.S., Nair S.V., Shanmugam M. (2018). 2D-Layered MoS2-Incorporated TiO_2_-Nanofiber-Based Dye-Sensitized Solar Cells. ChemistrySelect.

[23] Cheng C., Ren W., Zhang H. (2014). 3D TiO_2_/SnO_2_ hierarchically branched nanowires on transparent FTO substrate as photoanode for efficient water splitting. Nano Energy.

[24] Radecka M., Wnuk A., Trenczek-Zajac⁠ A., Schneider K., Zakrzewska⁠ K. (2015). TiO_2_/SnO_2_ nanotubes for hydrogen generation by photoelectrochemical water splitting. Int. J. Hydrogen Energy.

[25] Yuan W., Yuan J., Xie J., Li C.M. (2016). Polymer-Mediated Self-Assembly of TiO_2_@Cu_2_O Core-Shell Nanowire Array for Highly Efficient Photoelectrochemical Water Oxidation. ACS Appl. Mater. Interfaces.

[26] Yang Y., Xu D., Wu Q., Diao P. (2016). Cu_2_O/CuO bilayered composite as a high-efficiency photocathode for photoelectrochemical hydrogen evolution reaction. Sci. Rep..

[27] Zhang Q., Zhang K., Xu D., Yang G., Huang H., Nie F., Liu C., Yang S. (2014). CuO nanostructures: Synthesis, characterization, growth mechanisms, fundamental properties, and applications. Prog. Mater. Sci..

[28] Koiki B.A., Arotiba O.A. (2020). Cu_2_O as an emerging semiconductor in photocatalytic and photoelectrocatalytic treatment of water contaminated with organic substances: A review. RSC Adv..

[29] Sun S., Zhang X., Yang Q., Liang S., Zhang X., Yang Z. (2018). Cuprous oxide (Cu_2_O) crystals with tailored architectures: A comprehensive review on synthesis, fundamental properties, functional modifications and applications. Prog. Mater. Sci..

[30] Kusior A., Michalec K., Jelen P., Radecka M. (2019). Shaped Fe2O3 nanoparticles – Synthesis and enhanced photocatalytic degradation towards RhB. Appl. Surf. Sci..

[31] Lin Z., Li L., Yu L., Li W., Yang G. (2017). Dual-functional photocatalysis for hydrogen evolution from industrial wastewaters. Phys. Chem. Chem. Phys..

[32] Trenczek-Zajac A. (2019). Thermally oxidized CdS as a photoactive material. New J. Chem..

[33] Shen H., Zhao X., Duan L., Liu R., Wu H., Hou T., Jiang X., Gao H. (2017). Influence of interface combination of RGO-photosensitized SnO_2_@RGO core-shell structures on their photocatalytic performance. Appl. Surf. Sci..

[34] Ohsaka T., Izumi F., Fujiki Y. (1978). Raman spectrum of anatase, TiO_2_. J. Raman Spectrosc..

[35] Xu Y.H., Liang D.H., Liu M.L., Liu D. (2008). zhong Preparation and characterization of Cu_2_O-TiO_2_: Efficient photocatalytic degradation of methylene blue. Mater. Res. Bull..

[36] Ma Q., Zhang H., Cui Y., Deng X., Guo R., Cheng X., Xie M., Cheng Q. (2018). Fabrication of Cu_2_O/TiO_2_ nano-tube arrays photoelectrode and its enhanced photoelectrocatalytic performance for degradation of 2,4,6-trichlorophenol. J. Ind. Eng. Chem..

[37] Liu D., Tian R., Wang J., Nie E., Piao X., Li X., Sun Z. (2017). Photoelectrocatalytic degradation of methylene blue using F doped TiO_2_ photoelectrode under visible light irradiation. Chemosphere.

[38] da Silva M.R., Lucilha A.C., Afonso R., Dall’Antonia L.H., de Andrade Scalvi L.V. (2014). Photoelectrochemical properties of FTO/m-BiVO_4_ electrode in different electrolytes solutions under visible light irradiation. Ionics.

[39] Xiong L., Huang S., Yang X., Qiu M., Chen Z., Yu Y. (2011). P-Type and n-type Cu_2_O semiconductor thin films: Controllable preparation by simple solvothermal method and photoelectrochemical properties. Electrochim. Acta.

[40] Fernando C.A.N., De Silva P.H.C., Wethasinha S.K., Dharmadasa I.M., Delsol T., Simmonds M.C. (2002). Investigation of n-type Cu_2_O layers prepared by a low cost chemical method for use in photo-voltaic thin film solar cells. Renew. Energy.

[41] McShane C.M., Choi K.S. (2009). Photocurrent enhancement of n-type Cu_2_O electrodes achieved by controlling dendritic branching growth. J. Am. Chem. Soc..

[42] Aguirre E.M., Zhou R., Eugene A.J., Guzman M.I., Grela M.A. (2017). Cu_2_O/TiO_2_ heterostructures for CO_2_ reduction through a direct Z-scheme: Protecting Cu_2_O from photocorrosion. Appl. Catal. B Environ..

[43] Siripala W. (2008). Electrodeposition of n-type Cuprous Oxide Thin Films. ECS Trans..

[44] Garuthara R., Siripala W. (2006). Photoluminescence characterization of polycrystalline n-type Cu_2_O films. J. Lumin..

[45] Gerischer H., Seraphin B.O. (1979). Solar Photoelectrolysis with Semiconductor Electrodes. Solar Energy Conversion.

[46] Paracchino A., Laporte V., Sivula K., Grätzel M., Thimsen E. (2011). Highly active oxide photocathode for photoelectrochemical water reduction. Nat. Mater..

