# Supplementary material for: TiO2@Cu2O n-n Type Heterostructures for Photochemistry
Source: Materials (Basel). 2021 Jul 2;14(13):3725. doi: 10.3390/ma14133725 (PMC8269846; doi:10.3390/ma14133725)
Supplement: Supplementary file 1 [file materials-14-03725-s001.zip › materials-1265999 supplementary.pdf]

materials

Article

$TiO_2@Cu_2O$ n-n Type Heterostructures for Photochemistry

Anita Trenczek-Zajac [1,*], Joanna Banas-Gac [2] and Marta Radecka [1]

1 Department of Inorganic Chemistry, Faculty of Materials Science and Ceramics, AGH University of Science and Technology, al. A. Mickiewicza 30, 30-059 Krakow, Poland; radecka@agh.edu.pl
2 Institute of Electronics, Faculty of Computer Science, Electronics and Telecommunications, AGH University of Science and Technology, al. A. Mickiewicza 30, 30-059 Krakow, Poland; jbanas@agh.edu.pl
* Correspondence: anita.trenczek-zajac@agh.edu.pl; Tel.: +48-12-617-50-60

Using methylene blue requires some caution to be exercised. One should be aware that in the process of photocatalytic removal of the dye, it can be transformed into its colorless form—the leuco methylene blue (LMB) [1]—although due to the reaction kinetics, this process is not preferred [2]. It is, therefore, necessary to use a reducing agent in the form of, e.g., glucose [3], L-ascorbic acid [4–8], NaBH4, or metallic nanoparticles [2] to intentionally cause such a change. LMB can easily be oxidized to MB as a result of the following reaction [1]:

$$2LMB + O2 \rightarrow 2MB+ + 2OH- \quad (S1)$$

The above reaction is sensitive to the pH of the solution and with increasing pH its rate increases as well. According to Lee and Mills [1], leuco-methylene blue shows chemical stability—in darkness and in an air atmosphere—in a strongly acidic environment even below pH = 2. Under such conditions, it also retains some stability when it is being lightened. At pH > 2, the leuco form undergoes oxidation to a colorful MB (Equation (S1)) under the influence of lighting. In our work, the pH of the solution (MB + Na2SO4) in the process of photoelectrocatalytic decomposition of methylene blue was pH = 5.1, which indicates the fact that the oxidation of LMB according to Equation (S1) is possible. Therefore, even if some amount of MB was reduced to LMB, it could be oxidized back to MB, due to the presence of oxygen in the solution, acidic environment (pH > 2), and lighting. The presence of the reduced form LMB can be confirmed using the spectrophotometric method [1,8] and cyclic voltammetry [8,9]. To confirm or exclude the presence of methylene blue in the form of leuco in the solution, the first of them was chosen. The presence of LMB manifests itself by the absorption band at 256 nm next to the band arising from MB at 245 nm. The wavelength range in which the band appears was magnified (Figure S1) and shows no band from LMB, which excludes the presence of the leuco form of methylene blue in the solution of the dye after the process of photoelectrocatalysis.

Citation: Trenczek-Zajac, A.; Banas-Gac, J.; Radecka, M. $TiO_2@Cu_2O$ n-n Type Heterostructures for Photochemistry. *Materials* **2021**, *14*, 3725. https://doi.org/10.3390/ma14133725

Academic Editor: Víctor A. de la Peña O'Shea

Received: 2 June 2021
Accepted: 29 June 2021
Published: 2 July 2021

Publisher's Note: MDPI stays neutral with regard to jurisdictional claims in published maps and institutional affiliations.

Copyright: © 2021 by the authors. Licensee MDPI, Basel, Switzerland. This article is an open access article distributed under the terms and conditions of the Creative Commons Attribution (CC BY) license (http://creativecommons.org/licenses/by/4.0/)./by/4.0/).

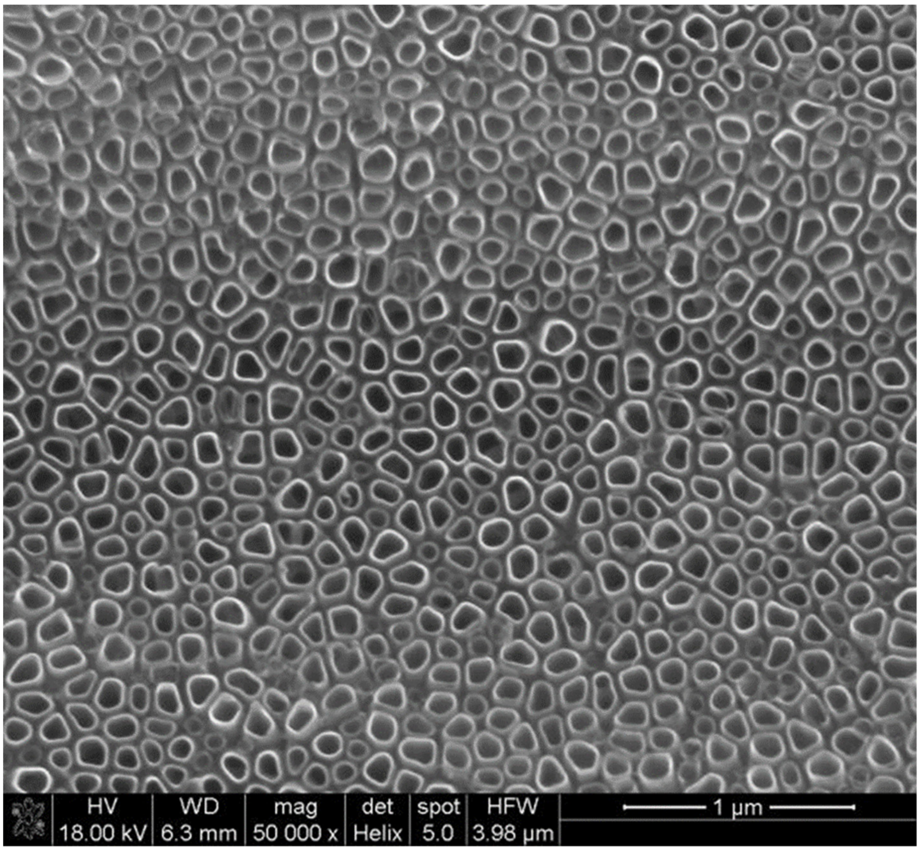

(**a**)

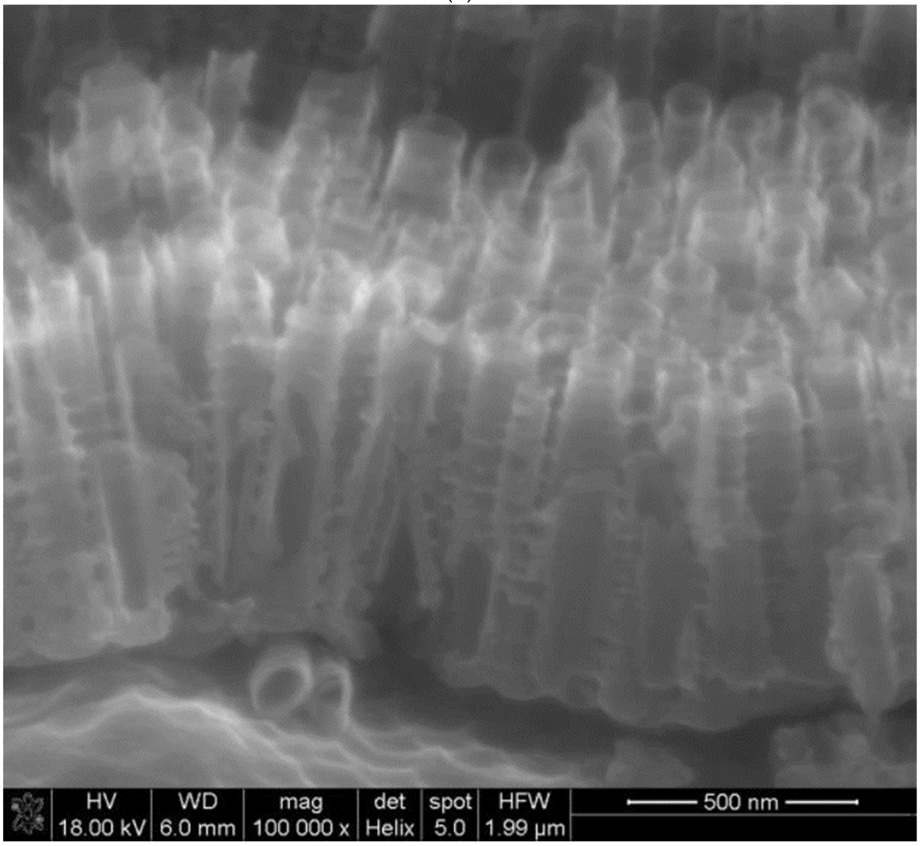

(**b**)

Figure S1. SEM images of TiO2-NT: (**a**) surface and (**b**) cross-section.

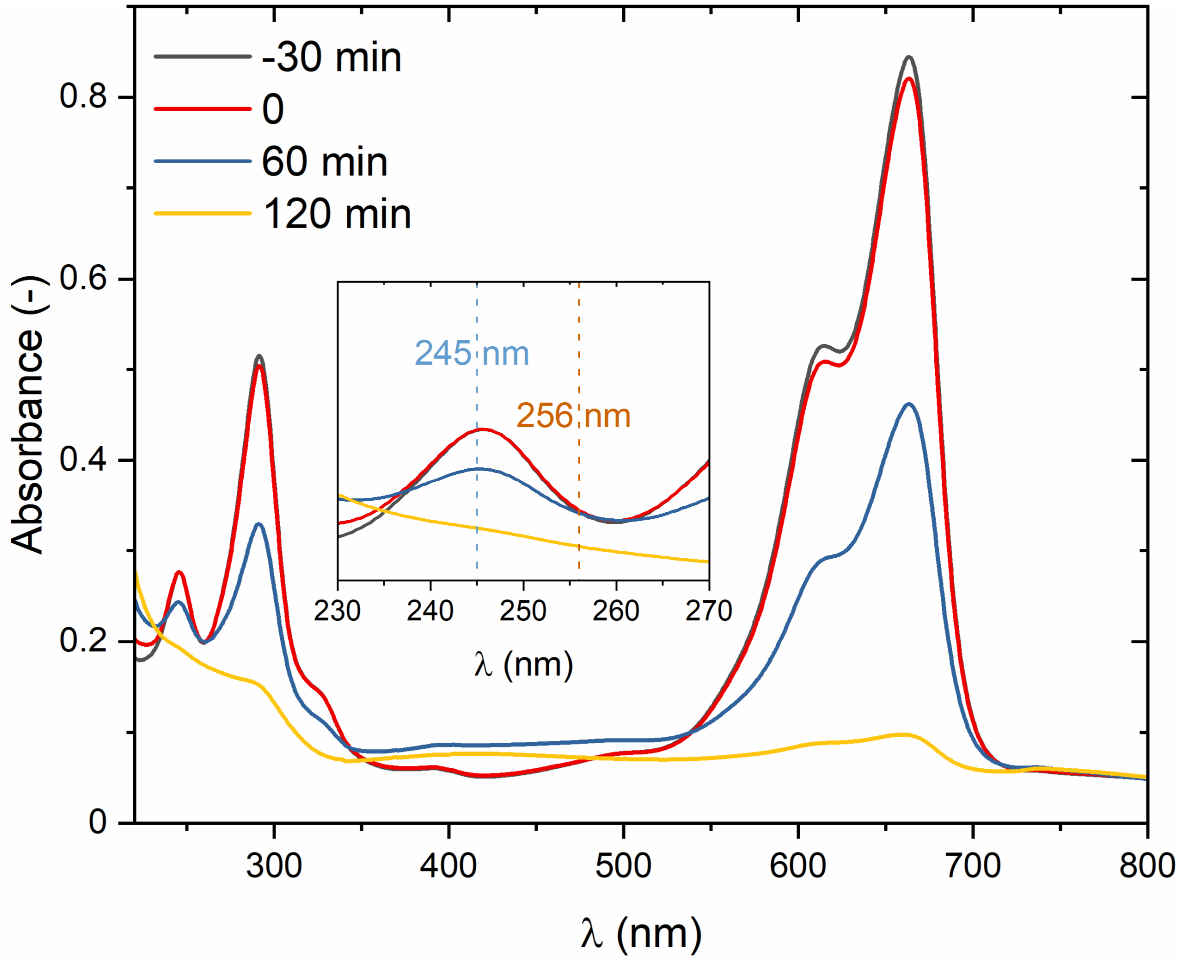

Figure S2. Absorption spectra of the MB + Na2SO4 mixture before and after photoelectrocatalysis of MB obtained for 5/NT heterostructure. Inset: position of the band derived from MB (blue) and LMB (orange).

References

1. Lee, S.-K.; Mills, A. Novel photochemistry of leuco-Methylene Blue. *Chem. Commun.* **2003**, *18*, 2366–2367, doi:10.1039/b307228b.
2. Begum, R.; Najeeb, J.; Sattar, A.; Naseem, K.; Irfan, A.; Al-Sehemi, A.G.; Farooqi, Z.H. Chemical reduction of methylene blue in the presence of nanocatalysts: a critical review. *Rev. Chem. Eng.* **2019**, *36*, 749–770, doi:10.1515/revce-2018-0047.
3. Gilbert Cook, A.; Tolliver, R.M.; Williams, J.E.; Cook, A.G. The blue bottle experiment revisited. How blue? How sweet? *J. Chem. Educ.* **1994**, *71*, 160–161, doi:10.1021/ed071p160.
4. Snehalatha, T.; Rajanna, K.C.; Saiprakash, P.K. Methylene Blue—Ascorbic Acid. An Undergraduate Experiment in Kinetics. *J. Chem. Educ.* **1997**, *74*, 228–233, doi:10.1021/ed074p228.
5. Mowry, S.; Ogren, P.J. Kinetics of Methylene Blue Reduction by Ascorbic Acid. *J. Chem. Educ.* **1999**, *76*, 970–973, doi:10.1021/ed076p970.
6. Leon, L.E. Amperometric flow-injection method for the assay of L-ascorbic acid based on the photochemical reduction of Methylene Blue. *Talanta* **1996**, *43*, 1275–1279, doi:10.1016/0039-9140(96)01846-2.
7. Sanz-Martinez, A.; Rios, A.; Valcarcelt, M. Photochemical Determination of Ascorbic Acid Using Unsegmented Flow Methods. *Analyst* **1992**, *117*, 1761–1765, doi:10.1039/AN9921701761.
8. White, V.R.; Fitzgerald, J.M. Continuous Determination of Ascorbic Acid by Photobleaching of Methylene Blue. *Anal. Chem.* **1972**, *44*, 1267–1269, doi:10.1021/ac60315a023.
9. Kosswattaarachchi, A.M.; Cook, T.R. Repurposing the Industrial Dye Methylene Blue as an Active Component for Redox Flow Batteries. *ChemElectroChem* **2018**, *5*, 3437–3442, doi:10.1002/celc.201801097.
